# Spontaneous penetration of gold nanoparticles through the blood brain barrier (BBB)

**DOI:** 10.1186/s12951-015-0133-1

**Published:** 2015-10-21

**Authors:** Hagit Sela, Hagit Cohen, Paz Elia, Raya Zach, Zeev Karpas, Yehuda Zeiri

**Affiliations:** Department of Biomedical Engineering, Ben-Gurion University of the Negev, Beer-Sheva, 8410501 Israel; Department of Chemistry, NRCN, P.O. Box 9001, Beer-Sheva, 8419001 Israel; The State of Israel Ministry of Health, Anxiety and Stress Research Unit, Faculty of Health Sciences, Beer-Sheva Mental Health Center, Ben-Gurion University of the Negev, Beer-Sheva, Israel

**Keywords:** Gold nanoparticles, Rat, Biokinetics, Penetration, Blood-brain barrier, ICP-MS, LA-ICP-MS

## Abstract

**Background:**

The blood brain barrier (BBB) controls the brain microenvironment and limits penetration of the central nervous system (CNS) by chemicals, thus creating an obstacle to many medical imaging and treatment procedures. Research efforts to identify viable routes of BBB penetration have focused on structures such as micelles, polymeric nanoparticles and liposomes as drug carriers, however, many of them failed to provide unequivocal proof of BBB penetration. Here we proved that gold nanoparticles (AuNPs) penetrate the BBB in rats to reach brain regions.

**Results:**

Injection of AuNPs to the abdominal cavity of rats resulted in levels of gold found in blood, urine, brain regions and body organs. After perfusion the concentration of gold in brain regions diminished dramatically indicating that most of the gold was in venous blood and not in the brain tissues. Injection of Na, K or Ca ion channel blockers reduced BBB penetration by half. A biological half-life of 12.9 ± 4.9 h was found for the gold nanoparticles. Possible mechanisms for the transport of AuNPs through the BBB are discussed.

**Conclusions:**

BBB penetration by AuNPs is spontaneous without the application of an external field. A major amount of gold resides in blood vessels therefore perfusion required. Ion channel blockers can be used to control the transport of AuNPs.

**Electronic supplementary material:**

The online version of this article (doi:10.1186/s12951-015-0133-1) contains supplementary material, which is available to authorized users.

## Background

Rigorous control of the brain microenvironment, essential to support neural signaling within the central nervous system (CNS), is provided by the blood-brain barrier (BBB). Present in all organisms with a developed CNS [1–3] the BBB not only prevents harmful agents from penetrating the CNS, it also blocks the delivery of drugs and therapeutics to the CNS. As such, the BBB has been an obstacle to the development of new drugs for the CNS. Indeed, most CNS drug candidates, essentially 100 % of large-molecule drugs and about 98 % of small-molecule drugs, cannot autonomously penetrate the BBB, and therefore, they also cannot access the brain [4].

Of the drugs that are able to cross the BBB, most do so via transmembrane diffusion, a mechanism that depends on the drug’s structural or chemical link to the cell membrane, and on integration or permeability through it. These phenomena, are mostly observed for low molecular weight and high lipid solubility are favored. Other molecular characteristics that can affect the partitioning of a drug molecule between the blood and the brain include molecular charge, tertiary structure and degree of protein binding [4]. In addition to transmembrane diffusion, some macromolecules, such as transferrin, are able to cross the BBB via a receptor-mediated process [5].

In the search for a viable approach to deliver therapeutics across the BBB, a number of prerequisites must be fulfilled. The process should be controllable and safe, with no negative effect on BBB integrity, and it should be biocompatible and selective [4–8]. Furthermore, as a method of target-specific drug delivery, it should deliver the drug to the site of intended action in the brain, where the drug load should be adequate such that therapeutic concentrations are obtained and maintained for sufficient time at the site targeted in the brain.

Numerous approaches of improving drug delivery to the brain have been reported [4–9]. Since BBB impenetrability is due to the presence of tight junctions (TJ) that prevent the passage of drug molecules, an obvious solution is to open the BBB in a controlled and reversible manner by disrupting its tight junctions, thereby enabling substances to infiltrate to the brain. To breach the tight junctions, various mechanisms such as the administration of osmotic solutions [10] or the application of a physical stimulus such as ultrasound [11] have been used. Different, noninvasive routes entailed the development of nanosystems such as liposomes, polymeric nanoparticles, solid lipid nanoparticles, polymeric micelles, and dendrimers that are able to deliver drugs to the brain. Administered intravenously, such colloidal drug carriers can leave the bloodstream and enter organs that possess porous endothelial capillaries. Despite the potential of these nanosystems, however, their insufficient BBB permeability confers on them only a limited capacity to transport therapeutics to the brain [5, 6, 9].

Perhaps the carrier method with the most promise entails gold nanoparticles (AuNPs), whose relevance extends beyond the transport of drugs to include other medicinal applications, such as imaging [12–14], radiotherapy of tumors [15, 16] and photo-thermal therapy [13]. In addition, AuNPs are also known for their ease of synthesis [17–19], simple functionalization procedures using therapeutic molecules [18, 19] and their good biocompatibility [20]. Taken together with their wide therapeutic applicability, the user-friendly, biocompatible nature of AuNPs makes them ideal candidates for BBB penetration.

The aims of the present study were to study the penetration of the BBB by gold nanoparticles and examine the effect of ion channel blockers on the transport of the AuNPs, as well as study the biokinetics of the distribution of the gold throughout several body organs and brain regions.

A proof for nanoparticle penetration through the BBB has been demonstrated by radiotherapy of brain tumors in mice [15, 16]. The present study provides unequivocal proof of spontaneous penetration of the BBB by AuNPs in the absence of an applied external field. Similar procedure to the one described here was used in Ref. [14].

## Results

We began our investigation by comparing the BBB penetrability of six different types of AuNPs distinguished by route of synthesis (see methods section). Five of the synthesized AuNPs were hydrophilic, and the sixth was hydrophobic due to a layer of perfluoro decanethiol (PFDT) adsorbed onto the particle surface. Whereas all the hydrophilic particles penetrated the BBB, the PFDT-functionalized particles did not. These results suggest that although the BBB is lipophilic in nature, hydrophilic AuNPs have higher probability to spontaneously penetrate the BBB. It should be noted that the AuNPs are covered by an organic layer that prevents them from agglomeration during their synthesis. When plant extracts were used as reducing agents, the detailed chemical nature of the organic layer is not fully identified, however, it contains many polar groups such as OH, NH and C=O (see Table 2 in Ref. [21]). The preliminary experiments with the six AuNPs suggested that those synthesized using *Pelargonium graveolens* (Rose Geranium) extract crossed the BBB with the highest efficiency, as discussed earlier [21] and in Additional file 1, hence, all the experiments reported below employed this type of AuNPs.

Our first goal was to provide proof that gold nanoparticles indeed penetrate the BBB. The brain is surrounded by, and contains, a very large number of blood vessels. Because the gold levels measured in the brain tissue are expected to be dominated by the gold content of the venous blood, a perfusion procedure was performed to remove venous blood from the brain tissues. After AuNP injection, gold concentrations in the different regions of the brain were measured with and without perfusion (Table 1).

**Table 1:** Gold concentrations in different brain regions following AuNP injection with and without perfusion

Organ	Perfusion (N = 4)	No perfusion (N = 2)
Frontal cortex	0.63 ± 0.27	6.21 ± 4.69
Hippocampus	0.56 ± 0.39	3.19 ± 3.45
Hypothalamus	0.31 ± 0.16^a^	4.56 ± 5.12

Gold concentrations (μg/g/mg gold injected) measured in different brain regions following AuNP injection with and without perfusion. To allow comparison between the results of the different experiments, the data were normalized to the amount of gold injected in each experiment

^a^A single high concentration value defined as an outlier was omitted. If this value is included the gold concentration found in the hypothalamus is 3.2 ± 5.2 μg/g/mg gold injected

In another experiment, AuNPs were injected abdominally into a rat. After 24 h the animal was sacrificed following perfusion, and its brain was frozen and dissected into 25 μm thick slices. A slice that contained the hippocampus and hypothalamus regions was selected for analysis via laser ablation inductively coupled plasma mass spectrometry (LA-ICP-MS). 2D images were constructed for the gold concentration distributions in the hypothalamus and in the hippocampus. The gold distribution in both brain regions was found to be practically uniform (see Additional file 2: Figure S2), suggesting that there were no preferred or selective penetration regions in these regions of the brain.

**Fig. 1:**
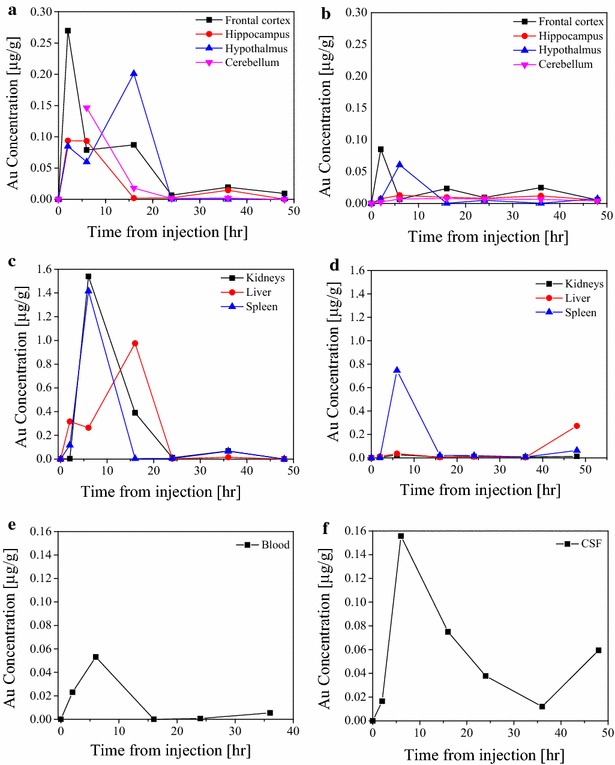
The biokinetic profiles of gold concentrations in different organs and regions of the brain as a function of the time after injection. Panels **a**, **c** and **e** correspond to the first set of experiments, while panels **b**, **d** and **f** correspond to the second set. **a** Variation of gold concentration in different brain regions in the first set of experiments. **b** Same as **a** for the second set of experiments. **c** Variation of gold concentration in internal organs in the first set of experiments. **d** Same as **c** for second set of experiments. **e** Variation of gold concentration in the blood in the first set of experiments. **f** Variation of gold concentration in the CSF in the second set of experiments

Next, the bio-kinetic profile of AuNPs in the rat body was studied. The profiles were obtained by sacrificing rats at predefined times after the injection of gold particles (2, 6, 16, 24, 36 and 48 h). After the rat was sacrificed, gold concentrations were measured in the kidney, liver, spleen and in brain regions (hippocampus, hypothalamus, frontal cortex and cerebellum). In many cases we could only extract part of the organ, hence, the concentration was determined as amount of Au in 1 gr of organ tissue. Two sets of experiments were performed, and for each, a freshly synthesized AuNP solution was prepared and used. In the first set of experiments the gold concentration in the solution was 105 mg/L while in the second set it was 60 mg/L The results are presented in Fig. 1. It should be noted that each data point in Fig. 1 corresponds to different rat.

To examine the importance of individual rat physiology, we compared gold concentrations in the urine of a single rat at different time points with the values obtained for other rats at the same time points (second set of experiments). The comparison results are summarized in Table 2.

**Table 2:** Comparison of gold concentrations in rat urine after injection of AuNPs

Time after AuNP injection (h)	Gold concentration in the urine of four different rats (mg L^−1^)	Gold concentration in the urine of a single rat (mg L^−1^)
2	0.057	0.013
6	0.158	0.110
16	0.027	Not examined
24	0.013	0.048

The final step in the present study was to assess the route through which the AuNPs penetrate the BBB. There are several possible mechanisms for penetration of nanoparticles through cell membranes including diffusion, direct penetration, via energy independent pathways and endocytosis [22]. The characteristics of the AuNPs used in this study, as discussed in previous work [21], exclude diffusion as the dominant mechanisms. The importance of direct penetration and penetration through specific transport sites was examined by blocking ion channels using dofetilide [23] and phenytoin [24] as K^+^ and Na^+^ ion channel blockers, respectively, and verapamil as a Ca^2+^ ion channel blocker [25]. Three sets of experiments with three rats in each set were carried out (i.e. total of nine rats). In each set freshly synthesized AuNP solutions were used for injection to each one of the three animals in the set according to the usual procedure described in the methods section below. One hour after AuNPs injection, one rat of the set was injected in its abdominal cavity with verapamil equal to 0.1 mg kg^−1^ rat body weight, a second rat was injected with a solution of dofetilide and phenytoin equal to 1 μg kg^−1^ and 8 mg kg^−1^ rat body weight, respectively. The third animal in each set served as control.

**Fig. 2:**
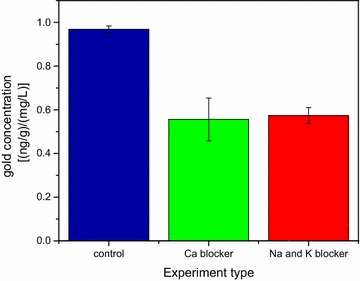
Change in gold concentrations in different brain regions due to exposure to ion channel blockers. Sum of gold concentrations measured in different brain regions as observed in the experiments with and without injection of the ion channel blockers (verapamil, dofetilide and phenytoin). Note that the concentration of the gold was normalized to the amount of gold nanoparticles in the solution that was injected in the different experiments

All the animals were sacrificed 3 h after the AuNPs injection. The concentrations of gold in three brain regions (frontal cortex, hippocampus and hypothalamus) were measured for each rat in the three sets examined. However, since the initial concentration of the AuNP solutions used for injection to each set had different Au concentration, the gold concentration measured in the various brain regions were normalized by the Au concentration in the solution used for the injection in each set. Some variations in gold concentration in the different brain regions were observed for the rats in each set. Since our main interest was in the overall influence of ion-channel blockers on the BBB permeation, the normalized gold concentration values, observed in the three brain regions in the three sets of experiments, were averaged. The average and standard deviation values obtained are presented in Fig. 2. The results shown in Fig. 2 clearly show that in the rats that were injected with ion channel blockers half the concentration of gold is found to cross the BBB compared to the corresponding value found in the control group.

## Discussion

With each heartbeat, arteries carry about 20–25 % of the total body blood to the brain. There are a large number of blood vessels surrounding the brain tissues and their structure is highly branched. Thus, the gold levels measured in the brain tissue are dominated by the gold content of the venous blood unless a perfusion procedure is carried out. The true level of gold in the brain tissues can be obtained after removal of venous blood to ensure that the measured gold level reflects the amount that penetrated the BBB.

The data in Table 1 show that the gold concentrations in all brain regions were significantly lower with perfusion than without it. The large post-perfusion reduction in gold concentration indicates that most of the gold measured without perfusion originated from the blood in the capillaries in the brain tissue and was not due to nanoparticle penetration of the BBB.

In a second set of experiments, cerebrospinal fluid (CSF) samples were collected after AuNPs injection and tested for the presence of gold. The average gold concentrations in CSF samples collected 6 h after injection was 0.16 mg L^−1^. Since the CSF is contained within a closed system surrounding the brain, the presence of gold in the CSF provides further evidence that AuNPs indeed penetrated the BBB.

Two sets of experiments were carried out in order to study the bio-kinetic behavior of the gold nanoparticles in the rat body. A comparison between the data obtained in the two experiments shows that the gold concentrations differed consistently by a factor of approximately five. This was attributed to the difference in size distribution of the AuNPs (about 98 % of which had a mean size of 1.3 ± 0.3 nm) in both preparations. This assumption is supported by the similarity in the profiles of the temporal behavior of the changes in gold concentration in the two experiments where, at 6–16 h after injection, the gold concentrations in the different brain regions reached their maximum levels.

Figure 1a and 1b show that the gold concentrations in the different brain regions varied during the 48 h after AuNP injection. Similar data are presented in Fig. 1c and 1d for the different organs examined. Note that technical difficulties in the first set of experiments prevented measurements of gold concentration in the cerebellum after 2 h. The picture that emerges from these results (Fig. 1a–d) is of a rapid initial increase in gold concentration to a maximum level after 6–16 h, followed by a fast decline until it levels out after about 24 h and slowly continues to decrease to a negligible level.

Considering the number and spacing of the data points, the maximum gold concentration cannot be determined more precisely. Even considering that each data point in Fig. 1 corresponds to a different animal that was sacrificed at that time the temporal profile indicates that individual differences in rat physiology had only a minor effect on the results. This conclusion was supported in complementary experiments designed to explore the urine kinetic profile of a single rat compared with the average values obtained for four other rats at the same time points (Table 2).

The gold concentration profiles in samples of blood (first set of experiments) and of CSF (second set of experiments) are shown (Fig. 1e and 1f, respectively). In both cases maximum gold concentration was obtained 6 h after AuNP injection, which subsequently decreased rapidly in both fluids. Because the AuNP solution is injected into the abdominal cavity of the animals, it is clear that its concentrations in different organs and brain regions are highly correlated with the gold concentration in the blood, the AuNP transport media.

In general, all brain regions and organs examined, as well as the CSF, demonstrated similar time dependent profiles variation of gold concentrations characterized by rapid increase in gold concentration during the first 10 h after injection followed by a decrease in concentration to low levels 24–48 h after injection. These variations are attributed to the variable gold concentration in the blood, which is the source of AuNP supply for all body organs, including the brain regions. Based on these results, the biological half-life of the gold nanoparticles in the rats’ bodies was estimated as 12.9 ± 4.9 h.

Possible pathways of the particle penetration through the BBB were investigated in order to complete the characterization study. The ability of nanoparticles to penetrate cell membrane was demonstrated by several research groups [26–31]. These studies clearly showed that the ability of nanoparticles to penetrate into cells is highly dependent on the type of cell and the particle characteristics such as size, surface charge and surface chemical composition. For example, it was confirmed that cationic nanoparticle have a much larger tendency to cross cell membrane compared to anionic nanoparticles [26, 27]. Since the gold nanoparticles used in this study had hydrophilic coating and were negatively charged (−23.9 ± 2 mV Zeta potential [21]), we anticipated that AuNP diffusion through membranes is not the dominant penetration mechanism. The BBB, as others barriers in the body, is composed of different tissue types. In the following discussion we considered the BBB as a uniform organ without addressing specifically different components.

An alternative BBB penetration mechanism entails the transport of AuNPs through more specific transport sites on the BBB. We examined the possibility for existence of such mechanism by blocking K^+^ and Na^+^ ion channels using dofetilide [23] and phenytoin [24], respectively, as ion channel blockers, and verapamil as a Ca^2+^ ion channel blocker [25]. The blockers were injected abdominally 1 h after the injection of AuNPs and the concentrations of gold in three brain regions (frontal cortex, hippocampus and hypothalamus) showed non-uniformity in the gold concentration between the three regions. However, the fact that in all three regions the gold content was markedly reduced compared to the concentrations found in the control rats clearly demonstrates that blocking the Ca^2+^, K^+^ and Na^+^ ion channels by either blocker led to marked reduction of close to 50 % in the total concentration of gold observed in the brain. This suggests that blocking ion channels can be used to control AuNPs penetration through the BBB.

The injection of ion channel blockers can influence AuNPs permeation in the whole body since they are expected to affect all ion channels in the body. The influence of ion channel blockers in internal organs, as the kidneys, may lead to reduced amount of AuNPs in the blood. Therefore, the reduced concentration of AuNPs found in the brain following injection of ion channel blockers may reflect a lower concentration of AuNPs in the blood. The results described here cannot differentiate between these two possibilities and it will be resolved in a future publication. Assuming that ion channel blockers mainly influence AuNPs permeation through the BBB, it can happen in two ways: either by reducing direct passage of AuNPs through the ion channels or by influencing the tight junctions (TJs). Notably the average diameters of Ca^2+^, Na^+^ and K^+^ ion channels is in the range 0.9–1.5 nm [32, 33] matches the average size of a large fraction of the AuNPs used in this study. Thus, one possible pathway is penetration of the BBB due to direct transport of small AuNPs through ion channels. However, it is known that the penetration through ion channels is highly affected and controlled by the affinity of the transported species to specific binding sites in the ion channel and not solely by size [34]. For example, although the ionic radius of Na^+^ is smaller than that of K^+^ its transport through K^+^ ion channels is prevented by the high specificity of the channel [34]. The specificity of the ion channels is expected to prevent transport of AuNPs through them. It is well established that the use of ion channel blockers leads to changes in ionic balance that result in a reduced permeability through the TJs [35, 36]. For example, variation in the extracellular calcium ion concentration is a critical component in tight junction regulation in models of Ca^2+^ addition/depletion [37–41]. Moreover, there are studies that connect migration through the BBB to changes in functionality due to blockage [42]. Hence, it is suggested that ion channel blockers can lead to changes in ion balance that are expressed in reduction of particle transfer through TJs.

## Conclusions

To the best of our knowledge, this is the first definitive study in which gold nanoparticles, synthesized using green chemistry approach, were shown to cross the BBB from the blood to various brain regions without the use of external fields. AuNP distributions in the hypothalamus and hippocampus were found to be nearly uniform (see Additional file 2). Injection of Ca^2+^ or Na^+^ and K^+^ ion channels blockers leads to 50 % reduction of gold concentrations in the brain. This finding suggests that either ion channels play a key role in the BBB permeability mechanism of small AuNPs or that the ion channel blockers influence the penetration of AuNPs through TJs due to changes in ion balance. The small diameter of ion channels and their high specificity suggests that reduced transport through TJs is probably the more important cause for the decreased gold concentration in the brain when ion channel blockers are used. If trans-membrane penetration of the endothelial cells constitutes an important route to cross the BBB, the addition of ion channel blockers must induce changes in the membrane that lead to reduced penetration efficiency of the AuNPs. This might also be related to the changes in ion balance induced by the ion channel blockers that lead to modifications in the membrane structure that result in reduced AuNPs penetration. This mechanism is supported by similar phenomenon described in the literature [35, 36]. The reduction in AuNPs concentration in the brain when ion channel blockers were injected can also be due to decrease in blood concentration of the gold nanoparticles as a result of changes in internal organ functionality when the ion channel blockers are present.

The bio-kinetics measurements showed that the temporal concentration profile was practically similar for all the body organs and brain regions examined and revealed that AuNPs in the rat body has a biological half-life of approximately 12.9 ± 4.9 h.

This study was aimed to examine the ability of AuNPs formed using green chemistry synthesis to penetrate through the BBB. Biomedical applications will require the encapsulation of drugs or diagnostic agents to the nanoparticles and might add to their size. However, since the diameter of the AuNPs used is about 2.5 nm, average size organic molecules used in many biomedical applications are much smaller and their addition is not expected to change significantly the particle size.

**Fig. 3:**
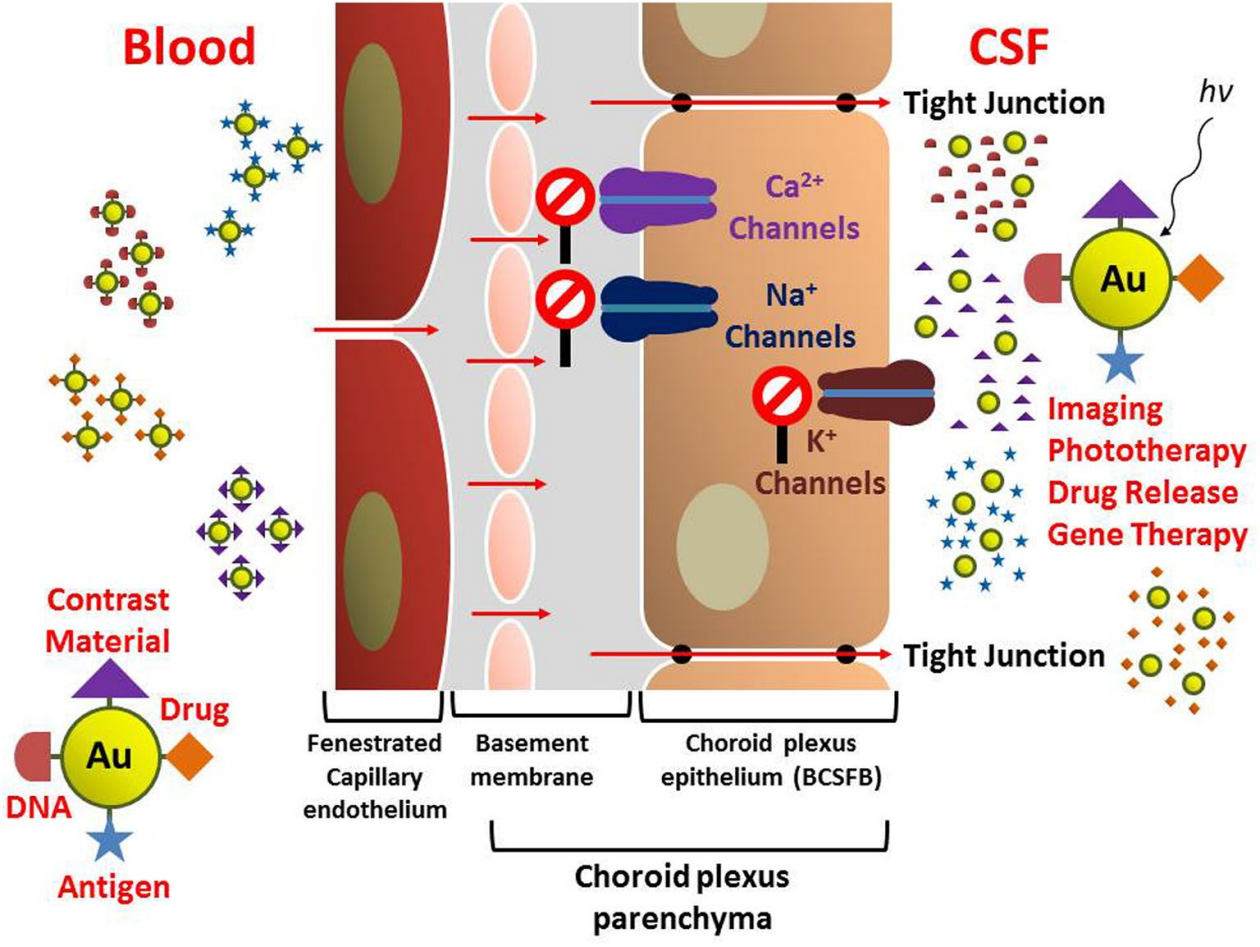
Schematic description of light-triggered biomedical applications using functionalized AuNPs that can penetrate the BBB

These findings suggest that the design of new and efficient drug delivery systems through the BBB could be based on the use of small AuNPs. The use of ion channel blockers allows one to have some control over the amount of AuNPs penetrating the BBB. Suitably functionalized AuNPs can also be used as imaging agents, for gene delivery or in photodynamic therapy applications as shown schematically in Fig. 3. These types of biomedical applications can be designed to be triggered by light at a wavelength suitable for desired interaction with the AuNPs that have crossed the BBB.

## Methods

AuNPs were produced via the reduction of HAuCl_4_ by several reducing agents, including sodium citrate [43] and the extracts of three common plants in Israel, *Salvia officinalis* (Common Sage), *Lippia citriodora* (Lemon Verbana) and *Pelargonium graveolens* (Rose Geranium). AuNP preparation and characterization using the plant extracts has been described in detail [21] and in Additional file 1. Another group of particles was produced by functionalizing particles obtained using sodium citrate as the reducing agent [43] and exposing them to 11-Mercapto-undecanoic acid (MUDA) or to Perfluoro Decanethiol (PFDT) to produce hydrophilic or hydrophobic AuNPs, respectively. The size distributions of all AuNPs were measured using dynamic light scattering (DLS). Normalization of the DLS data according to particle numbers showed that in all cases the average diameter size of the AuNP particles synthesized using *Pelargonium graveolens* was in the range of 1.3 ± 0.3 nm with Zeta potential −23.9 ± 2 mV (all error quantities described in this study correspond to SD values) [21]. However, the concentration of the gold in the injected solution was in the range of 60–120 mg L^−1^ as measured by ICP-MS.

Male Sprague Dawley rats weighing 200–250 g were habituated to housing conditions for at least seven days. The rats were housed four per cage in a vivarium with stable temperature and a reversed 12-h light/dark cycle and with unlimited access to food and water. A volume of 200 µL of solution containing gold nanoparticles was injected into the abdominal cavity of the rats (total of 12–24 μg Au in each injection depending on the initial Au concentration).

The health conditions of the rats that followed the injection indicate for normal locomotor activity (without stereotypical behavior, e.g., repeatedly chewing metal cage bars), normal eating and drinking, cleaned, normal posture, without anxiety and aggression; no vocalisation.

The accumulated amount of gold was determined in various regions in the brain (i.e., hippocampus, frontal cortex, entire cortex, cerebellum and hypothalamus) and in different internal organs (kidneys, liver and spleen). Urine samples were collected when it was possible about 2 h after injection and at the end of the experiments. For CSF collection, the animals were anesthetized (ketamine and xylazine mixture) and fixed in a stereotactic device (for details see Additional file 3). CSF sample volume ranged from 100–150 µL. Blood samples were collected when the animal was sacrificed.

Perfusion protocol: animals were deeply anesthetized (ketamine and xylazine mixture) and perfused transcardially with cold 0.9 % physiological saline followed by 4 % paraformaldehyde (Sigma-Aldrich) in 0.1 mol/L phosphate buffer (pH 7.4) 220 and 200 mL respectively. Brains were quickly removed, post fixed in the same fixative for 12 h at 4 °C, and were cryo-protected overnight in 30 % sucrose in 0.1 mol/L phosphate buffer at 4 °C. the procedure last 45–60 min.

AuNP bio-kinetics was determined by monitoring the gold concentrations in different body organs at different times after AuNP injection. As such, the gold concentrations in the different organs were determined for different rats, where the number of participating animals was equal to the number of time periods examined. All the animals were injected with 200 µL of the AuNP solution to their abdominal cavity. Animals were sacrificed at predetermined times and the gold concentrations in their organs were examined. Samples were collected at 2, 6, 16, 24, 36 and 48 h following injection. After the perfusion was performed, all the organs were removed and preserved for subsequent analysis of gold concentration. In addition, blood and urine samples were also collected for most of the examined objects.

Verapamil, dofetilide and phenytoin were used as a Ca^2+^, K^+^ and Na^+^ ion channel blockers, respectively, to examine their effect on the BBB penetration of AuNPs. In these experiments rats were injected with 0.1 mg kg^−1^ body weight of Verapamil, or a solution of 1 μg kg^−1^ body weight of dofetilide and 8 mg kg^−1^ body weight of phenytoin 1 h after the injection of the AuNPs. These experiments were terminated by sacrificing the animals 3 h following AuNP injection.

Instrumentation: A quadrupole-based Inductively Coupled Plasma-Mass Spectrometer (ICP-MS, ELAN DRC-e, Perkin-Elmer Sciex) was used for all elemental analyses. The ICP-MS was also coupled to a laser ablation (LA-ICP-MS) system (CETAC LSX-213, CETAC Technologies, Inc., Omaha, NE, USA) that enabled the gold spatial distribution in slices of rat brain to be characterized and imaged. These analyses were carried out at the Geological Survey of Israel (GSI), Jerusalem. For details of the parameters used see Additional file 4.

## Additional files

10.1186/s12951-015-0133-1 Title of data: Synthesis and characterization of the gold nanoparticles (AuNPs). Description of the preparation procedure of the AuNPs and Analysis of the matching levels of the different particles to our experiments purposes.
10.1186/s12951-015-0133-1 LA-ICP-MS 2D imaging of gold distribution in the hippocampus and the hypothalamus. Figure S1 present LA-ICP-MS 2D imaging of gold distribution in the hippocampus and the hypothalamus, as representative regions of brain.
10.1186/s12951-015-0133-1 CSF collection procedure. Description of the CSF collection procedure.
10.1186/s12951-015-0133-1 Instrument optimization. Tables S1 and S2 summarize the optimal operational parameters for the Inductively Coupled Plasma-Mass Spectrometry (ICP-MS) and Laser Ablation-ICP-MS systems used in the present work.

## Abbreviations

AuNPs: Gold nanoparticles
BBB: Blood brain barrier
CNS: Central nervous system
CSF: Cerebrospinal fluid
LA-ICP-MS: Laser ablation inductively coupled plasma mass spectrometry
MUDA: 11-Mercapto-undecanoic acid
PFDT: Perfluoro decanethiol
