# Supplementary material for: Spontaneous penetration of gold nanoparticles through the blood brain barrier (BBB)
Source: J Nanobiotechnology. 2015 Oct 21;13:71. doi: 10.1186/s12951-015-0133-1 (PMC4618365; doi:10.1186/s12951-015-0133-1)
Supplement: Supplementary file 1 — 10.1186/s12951-015-0133-1 Title of data: Synthesis and characterization of the gold nanoparticles (AuNPs). Description of the preparation procedure of the AuNPs and Analysis of the matching levels of the different particles to our experiments purposes. [file 12951_2015_133_MOESM1_ESM.docx]

**Synthesis and characterization of the gold nanoparticles (AuNPs)**

The permeability of gold nanoparticles, synthesized by different procedures, through the blood-brain barrier (BBB) into different regions of the brain (hippocampus, frontal cortex and hypothalamus) was examined.

Six different preparation procedures were used. First, a protocol based on sodium citrate as a reducing agent as suggested by G. Frens [1] ("Classic"). The second, third and fourth protocols, were also based on G. Frens [2] but using herbal extracts as reducing agents in the synthesis of the gold nanoparticles. Three types of extracts were used: from the Salvia, Rose Geranium and Aloysia plants and the produced particles were referred to as "Salvia", "Geranium" and "Aloysia", respectively. The herbs were selected on the basis of their well-known compatibility with the human body since they are all used in herbal medicines. The fifth and sixth protocols, based on functionalizing the "Classic" particle surface using 11-Mercapto-undecanoic acid (MUDA) as a hydrophilic functional group, and Perfluoro Decanethiol (PFDT) as a hydrophobic functional group. In order to examine the efficiency of gold nanoparticles penetration to the brain a perfusion procedure must be carried out. This procedure is complicated and time consuming, hence, it was not performed in the preliminary experiments whose results were used to select the reducing agent to be used in the study reported here.

Figure 2S presents the amount of gold found in three different brain regions 24 hours after injection of the six different types of gold nanoparticles solutions. There is no clear pattern of the gold particles distribution in the different brain regions although the observed gold concentration in the hypothalamus is higher than in the other regions. In general, examining the overall penetration of AuNPs to the brain, the Classic, PFDT and MUDA types of particles appear to be less efficient in penetrating the BBB. It is not surprising that the PFDT particles show the lowest degree of penetration since they have a hydrophobic nature and the literature provides several examples of particle-cell interaction, emphasizing the importance of parameters such as particle charge and hydrophilicity [2-4]. Moreover, the hydrophobic nature of the PFDT functionalized particle limits the concentration of the AuNPs to small values. Higher BBB permeability is clearly associated with gold particles synthesized by the three green chemistry protocols. The AuNPs prepared using Geranium extracts, were chosen to be used in the present study due to three reasons: its ease of synthesis, its good overall performance as seen in Fig. S1 and its smallest diameter as observed in the DLS measurements[18].

**Figure S1:** Gold distribution in three brain regions for gold nanoparticles obtained by the six preparations described in the text.

(1) Frens G. Controlled nucleation for the regulation of the particle size in monodisperse gold solutions. *Nat.Phys.Sci.* 241, 20-22 (1973).

(2) Sun X, Rossin R, Turner J., et al. An assessment of the effects of shell cross-linked nanoparticle size, core composition, and surface PEGylation on in vivo biodistribution. *Biomacromolecules.* 6, 2541–2554 (2005).

(3) Avgoustakis K, Beletsi A, Panagi Z, et al. Effect of copolymer composition on the physicochemical characteristics, in vitro stability, and biodistribution of PLGA–mPEG nanoparticles. *Int. J. Pharm.* 259, 115–127 (2003).

(4) Illum L, Davis SS, Muller R.H, Mak E, West P, The organ distribution and circulation time of intravenously injected colloidal carriers sterically stabilized with a block copolymer – poloxamine 908. Life Sci. 40, 367–374 (1987)*.*
