# Supplementary material for: Spontaneous penetration of gold nanoparticles through the blood brain barrier (BBB)
Source: J Nanobiotechnology. 2015 Oct 21;13:71. doi: 10.1186/s12951-015-0133-1 (PMC4618365; doi:10.1186/s12951-015-0133-1)
Supplement: Supplementary file 2 — 10.1186/s12951-015-0133-1 LA-ICP-MS 2D imaging of gold distribution in the hippocampus and the hypothalamus. Figure S1 present LA-ICP-MS 2D imaging of gold distribution in the hippocampus and the hypothalamus, as representative regions of brain. [file 12951_2015_133_MOESM2_ESM.docx]

**LA-ICP-MS 2D imaging of gold distribution in the hippocampus and the hypothalamus**

Figure S2 present LA-ICP-MS 2D imaging of gold distribution in the hippocampus and the hypothalamus, as representative regions of brain. The images obtained from a brain sample that was frozen and dissected into 25 μm thick slices after AuNPs abdominal injection.


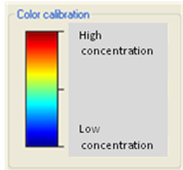


Low Concentration

High Concentration

| 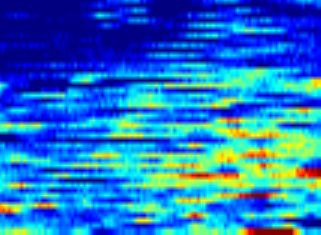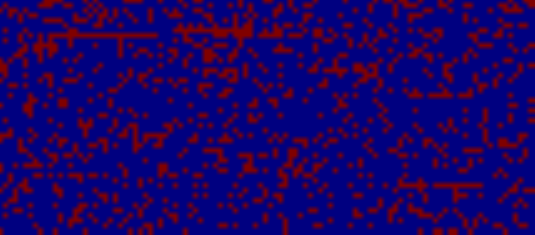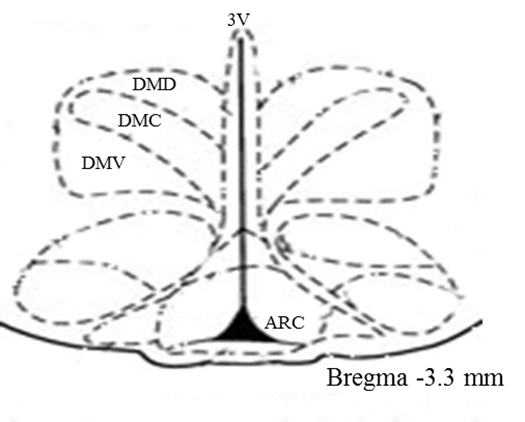  **A**  **C**  **E** | 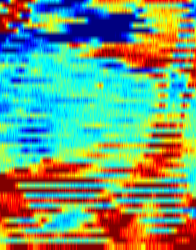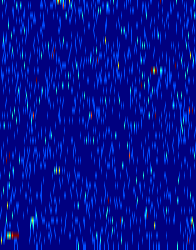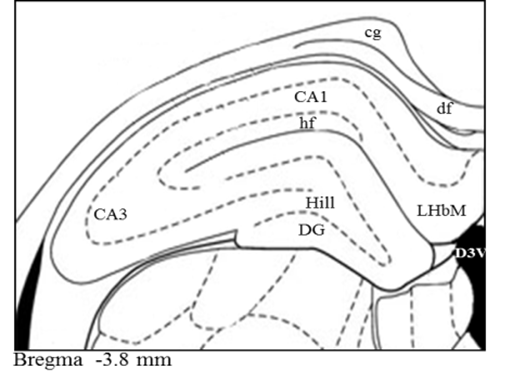  **D**  **B**  **F** |
| --- | --- |

Figure S2: Qualitative LA-ICP-MS imaging of gold distribution in rat brain sections. (**A)** and (**B**) atlas drawings of the hypothalamus and hippocampus respectively [1]; (**C**) Carbon-13 distribution in the hypothalamus; (**D**) Carbon-13 distribution in the hippocampus; (**E**) Gold distribution in the hypothalamus; (**F**) Gold distribution in the hippocampus.

(1) Paxinos G, Watson C. The Rat Brain in Stereotaxic Coordinates: London, UK (1988).
