# Supplementary material for: Spontaneous penetration of gold nanoparticles through the blood brain barrier (BBB)
Source: J Nanobiotechnology. 2015 Oct 21;13:71. doi: 10.1186/s12951-015-0133-1 (PMC4618365; doi:10.1186/s12951-015-0133-1)
Supplement: Supplementary file 3 — 10.1186/s12951-015-0133-1 CSF collection procedure. Description of the CSF collection procedure. [file 12951_2015_133_MOESM3_ESM.docx]

**CSF collection procedure**

The angle between the head of the rat and its body was set at 90° as shown in Figure S3; punctured surfaces were positioned horizontally. The skin from spinous process projecting at the initial part of the vertebral column and skull bones was cut along the median line (10-15 mm). The tissues were mechanically displaced from the midline of muscular fasciae on the neck until dura mater appeared. The dura mater looked like a stretched membrane and had mat surface (rhombus). The parietal bone, vertebral column, and occipital protuberances formed the cerebral, caudal, and lateral corners of the rhombus, respectively. The membrane was perpendicularly punctured at the middle point of the midline (depth 1 mm). The maximum amount of the liquor was sampled. The needle was removed. A median longitudinal incision (1.5 mm) was made through the site of puncture in the dura mater. The residual liquor was removed from cisterna magna (CM) with a cotton plug. The cavity of CM was filled with freshly prepared acryl emulsion using a micropipette. After induration of acryl, CM models were separated from surrounding tissues and examined in transmitted light. Profiles of CM were visualized on the screen and sketched in the frontal and sagittal surfaces. The actual size of CM was measured on models with a micrometer [1-3].


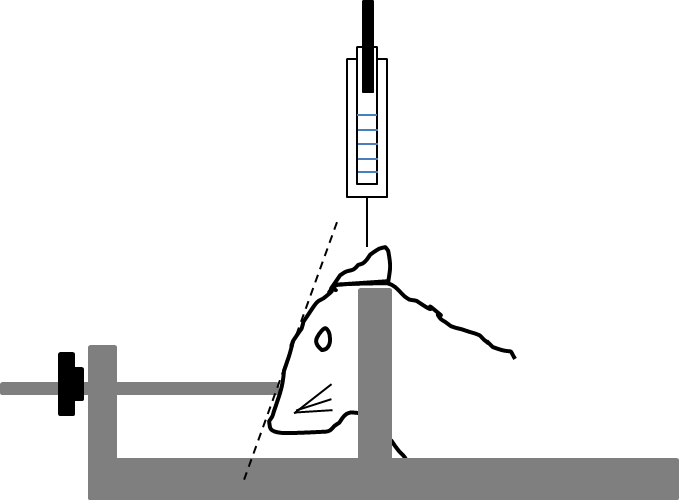


**Figure S3.** Fixation of rats in a stereotactic device during puncture of the great cerebral cistern[1].

(1) Lebedev SV, Blinov DV, Petrov SV, Spatial Characteristics of Cisterna Magna

in Rats and Novel Technique for Puncture with a Stereotactic Manipulator. Bulletin of Experimental Biology and Medicine, 137(6), 635-638 (2004).

(2)[Mahat MY](http://www.ncbi.nlm.nih.gov/pubmed?term=Mahat%20MY%5BAuthor%5D&cauthor=true&cauthor_uid=23000275), [Fakrudeen Ali Ahamed N](http://www.ncbi.nlm.nih.gov/pubmed?term=Fakrudeen%20Ali%20Ahamed%20N%5BAuthor%5D&cauthor=true&cauthor_uid=23000275), [Chandrasekaran S](http://www.ncbi.nlm.nih.gov/pubmed?term=Chandrasekaran%20S%5BAuthor%5D&cauthor=true&cauthor_uid=23000275), [Rajagopal S](http://www.ncbi.nlm.nih.gov/pubmed?term=Rajagopal%20S%5BAuthor%5D&cauthor=true&cauthor_uid=23000275), [Narayanan S](http://www.ncbi.nlm.nih.gov/pubmed?term=Narayanan%20S%5BAuthor%5D&cauthor=true&cauthor_uid=23000275), [Surendran N](http://www.ncbi.nlm.nih.gov/pubmed?term=Surendran%20N%5BAuthor%5D&cauthor=true&cauthor_uid=23000275). An improved method of transcutaneous cisterna magna puncture for cerebrospinal fluid sampling in rats. [J Neurosci Methods.](http://www.ncbi.nlm.nih.gov/pubmed/23000275) 15;211(2):272-279 (2012).

(3) Pegg CC, He C, Stroink AR, Kattner KA, Xu Wang, C, [Technique for collection of cerebrospinal fluid from the cisterna magna in rat](http://www.sciencedirect.com/science/article/pii/S0165027009006244). Journal of Neuroscience Methods, 187, (1), 8–12 (2010).
