# Supplementary material for: Spontaneous penetration of gold nanoparticles through the blood brain barrier (BBB)
Source: J Nanobiotechnology. 2015 Oct 21;13:71. doi: 10.1186/s12951-015-0133-1 (PMC4618365; doi:10.1186/s12951-015-0133-1)
Supplement: Supplementary file 4 — 10.1186/s12951-015-0133-1 Instrument optimization. Tables S1 and S2 summarize the optimal operational parameters for the Inductively Coupled Plasma-Mass Spectrometry (ICP-MS) and Laser Ablation-ICP-MS systems used in the present work. [file 12951_2015_133_MOESM4_ESM.docx]

**Instrument optimization**

Tables S1 - S2 summarize the optimal operational parameters for the Inductively Coupled Plasma-Mass Spectrometry (ICP-MS) and Laser Ablation-ICP-MS systems used in the present work.

**Table S1: Optimal operating parameters of wet plasma experimental systems**

| ICP-MS |  |
| --- | --- |
|  | Perkin Elmer ELAN DRC-e |
| Plasma conditions |  |
| Incident RF power | 1150 Watts |
| Cooling gas flow rate | Ar 10.0 L min^−1^ |
| Auxiliary gas flow rate | Ar 0.95 L min^−1^ |
| Nebulizer gas flow rate | Ar 0.70 L min^−1^ |
| Detection mode | Dual |
| Auto lens | On |
| Scanning mode | Peak hopping |

**Table S2: Optimized operating parameters of the dry plasma and laser ablation experimental systems**

| ICP-MS |  |
| --- | --- |
|  | Perkin Elmer ELAN DRC-e |
| Plasma conditions |  |
| Incident RF power | 1150 Watts |
| Cooling gas flow rate | Ar 10.0 L min^-1^ |
| Auxiliary gas flow rate | Ar 0.95 L min^-1^ |
| Nebulizer gas flow rate | Ar 0.70 L min^-1^ |
| Detection mode | Dual |
| Auto lens | On |
| Scanning mode | Peak hopping |

| Laser Ablation |  |
| --- | --- |
|  |  |
| Laser ablation parameters |  |
| Laser | Nd:YAG |
| Wavelength | 213 nm |
| Repetition rate | 20 Hz |
| Energy | 750 Volt |
| Spot size | 25 μm |
| Scan rate | 40 μm sec^-1^ |
| Carrier gas flow | 0.4 L min^-1^ |
